# Bevacizumab Treatment of Radiation-Induced Brain Necrosis: A Systematic Review

**DOI:** 10.3389/fonc.2021.593449

**Published:** 2021-03-25

**Authors:** Guixiang Liao, Muhammad Khan, Zhihong Zhao, Sumbal Arooj, Maosheng Yan, Xianming Li

**Affiliations:** ^1^ Department of Radiation Oncology, Shenzhen People’s Hospital, The First Affiliated Hospital of Southern University of Science and Technology, Shenzhen, China; ^2^ Department of Oncology, First Affiliated Hospital of Anhui Medical University, Hefei, China; ^3^ Department of Nephrology, Shenzhen People’s Hospital, Second Clinical Medicine Centre, Jinan University, Shenzhen, China; ^4^ Department of Biochemistry, University of Sialkot, Sialkot, Pakistan

**Keywords:** bevacizumab (BV), radiation-induced brain necrosis (RBN), dexamethasone, neurocognition, magnetic resonance imaging (MRI), adverse events

## Abstract

**Background:**

Radiation brain necrosis (RBN) is a serious complication in patients receiving radiotherapy for intracranial disease. Many studies have investigated the efficacy and safety of bevacizumab in patients with RBN. In the present study, we systematically reviewed the medical literature for studies reporting the efficacy and safety of bevacizumab, as well as for studies comparing bevacizumab with corticosteroids.

**Materials and Methods:**

We searched PubMed, Cochrane library, EMBASE, and ClinicalTrials.gov from their inception through 1 March, 2020 for studies that evaluated the efficacy and safety of bevacizumab in patients with RBN. Two investigators independently performed the study selection, data extraction, and data synthesis.

**Results:**

Overall, the present systematic review included 12 studies (eight retrospective, two prospective, and two randomized control trials [RCTs]) involving 236 patients with RBN treated who were treated with bevacizumab. The two RCTs also had control arms comprising patients with RBN who were treated with corticosteroids/placebo (n=57). Radiographic responses were recorded in 84.7% (200/236) of patients, and radiographic progression was observed in 15.3% (36/236). Clinical improvement was observed in 91% (n=127) of responding patients among seven studies (n=113). All 12 studies reported volume reduction on T1 gadolinium enhancement MRI (median: 50%, range: 26%–80%) and/or T2 FLAIR MRI images (median: 59%, range: 48%–74%). In total, 46 responding patients (34%) had recurrence. The two RCTs revealed significantly improved radiographic response in patients treated with bevacizumab (Levin et al.: p = 0.0013; Xu et al.: p < 0.001). Both also showed clinical improvement (Levin et al.: NA; Xu et al.: p = 0.039) and significant reduction in edema volume on both T1 gadolinium enhancement MRI (Levin et al.: p=0.0058; Xu et al.: p=0.027) and T2 FLAIR MRI (Levin et al.: p=0.0149; Xu et al.: p < 0.001). Neurocognitive improvement was significantly better after 2 months of treatment in patients receiving bevacizumab than in those given corticosteroids, as assessed by the MoCA scale (p = 0.028). The recurrence rate and side effects of the treatments showed no significant differences.

**Conclusions:**

Patients with RBN respond to bevacizumab, which can improve clinical outcomes and cognitive function. Bevacizumab appears to be more efficacious than corticosteroid-based treatment. The safety profile was comparable to that of the corticosteroids.

## Introduction

Radiotherapy is widely used to treat intracranial diseases (1, 2). However, patients receiving radiotherapy in the brain often present with a late complication called radiation-induced brain injury (RIBI) (3). RIBI is categorized as acute (days to weeks after irradiation), early delayed (1–6 months after irradiation), or late delayed (> 6 months after irradiation) (4). Advancements in immunotherapy have greatly increased the survival rates of patients with brain disease, as have the combination of radiotherapy with immune checkpoint inhibitors or targeted therapy agents (5). As a result, patients are more at risk of experiencing late delayed brain injury, characterized histopathologically by vascular abnormalities, demyelination, and ultimately white matter necrosis (6). Radiation-induced brain necrosis (RBN) is one of the main limiting toxicities, generally occurring 6 months to several years after treatment (7). Furthermore, combining radiation with targeted/immunotherapeutic agents to treat metastatic brain disease confers an increased risk of RBN that must be weighed against the synergistic effects of the treatment (8, 9). Therefore, the diagnosis and treatment of RBN are a crucial element in the management of patients with brain diseases.

The pathogenesis of RBN involves late barrier dysfunction and post-irradiation hypoxia, which leads to upregulation of vascular endothelial growth factor (VEGF) (10, 11). More specifically, radiotherapy induces endothelial cell loss through acid sphingomyelinase-dependent apoptosis, causing vasogenic edema, ischemia, and hypoxia. As a result, hypoxia-inducible factor 1α (HIF1α) is upregulated, leading to increased VEGF production in astrocytes and endothelial cells, mainly in the white matter around areas of necrosis (10). Increased levels of VEGF in reactive astrocytes around a core of necrotic tissue have also been observed using immunohistochemistry in surgical samples of RBN (11), suggesting that VEGF plays a critical role in the development of RBN, and that targeting VEGF with anti-VEGF antibodies could halt RBN development. For this reason, such treatment has been the main focus of preclinical and clinical research in recent years.

Several studies have assessed the efficacy and safety of bevacizumab, an anti-VEGF antibody, in the treatment of RBN. Most of these were case reports or retrospective studies with very few patients and lacked radiographic/clinical evidence (12–19). In the present study, we systematically reviewed the literature for studies investigating the efficacy and safety of bevacizumab in the treatment of RBN. We also searched for studies that compared bevacizumab with corticosteroids in this regard.

## Materials and Methods

### Study Registration

This systematic review complied with the PRISMA statement. The protocol was registered on PROSPERO: CRD42019134033.

### Criteria for Considering Studies

#### Study Types

This systematic review included published studies with retrospective, prospective, or randomized controlled trial research designs and more than five participants. Single-arm studies investigating the efficacy and safety of bevacizumab in the treatment of RBN were included, as were studies with comparative arms comparing bevacizumab to corticosteroids in this regard. Other types of studies, such as case reports, conference presentations, comments, studies with fewer than five participants, and studies lacking radiographic evidence were excluded.

#### Types of Patients

All studies involved patients with RBN who were aged ≥ 18 years old.

#### Types of Interventions

In all studies, bevacizumab was used to treat RBN. In some, its efficacy and safety were compared with those of corticosteroids. Other control interventions included placebo, other pharmacological treatments, hyperbaric oxygen, or laser interstitial thermal therapy.

#### Outcomes of Interest

The primary outcomes included clinical improvement, assessed in terms of symptomatic improvement/resolution, increase in Karnofsky performance status (KPS) score, decrease in dexamethasone use, radiographic response, and edema volume changes under T2-weighted FLAIR MRI images or T1-weighted gadolinium (Gd)-enhanced MRI images. Secondary outcomes of interest included rates of necrosis recurrence, rates of adverse effects, and cognitive function.

### Searching Methods for Eligible Studies

The following databases were included in the search: PubMed, Cochrane Library, EMBASE, and ClinicalTrials.gov since their inception. The latest search was conducted on March 1, 2020. The language was restricted to English. The main search terms were “radiotherapy,” “radiation brain necrosis,” and “bevacizumab.” References from all the identified studies were also examined for any additional relevant randomized control trials (RCTs).

### Selection of Studies

The studies from the initial search were imported into the NoteExpress software; all duplicate references were excluded. The remaining studies underwent title and abstract screening by two independent authors (G.L. and Z.Z.). The studies that met the inclusion criteria were retrieved in full-text form and further screened and evaluated by two independent reviewers for final inclusion. Any disagreement was discussed and resolved by all authors.

### Data Extraction

Data from the included studies were extracted using a pre-piloted and standardized method. The following information was extracted: first author of the study, country location, year of publication, sample sizes for each intervention, details of intervention methods, details of patients in each intervention (age, sex, cancer type, dose, and duration of radiation), and outcomes of interest (response rate, progression disease, volume changes of edema under MRI, cognitive function, and adverse effects). Data were extracted by two independent reviewers. Any dispute was resolved through discussion by all reviewers.

### Quality and Risk of Bias Assessment

A reporting checklist created by the MOOSE group was used to assess the quality of the retrospective studies (20). The risk of bias for RCTs was evaluated using Cochrane tools by a pair of reviewers (G.L. and H.Z.) (21).

## Results

**Figure 1:**
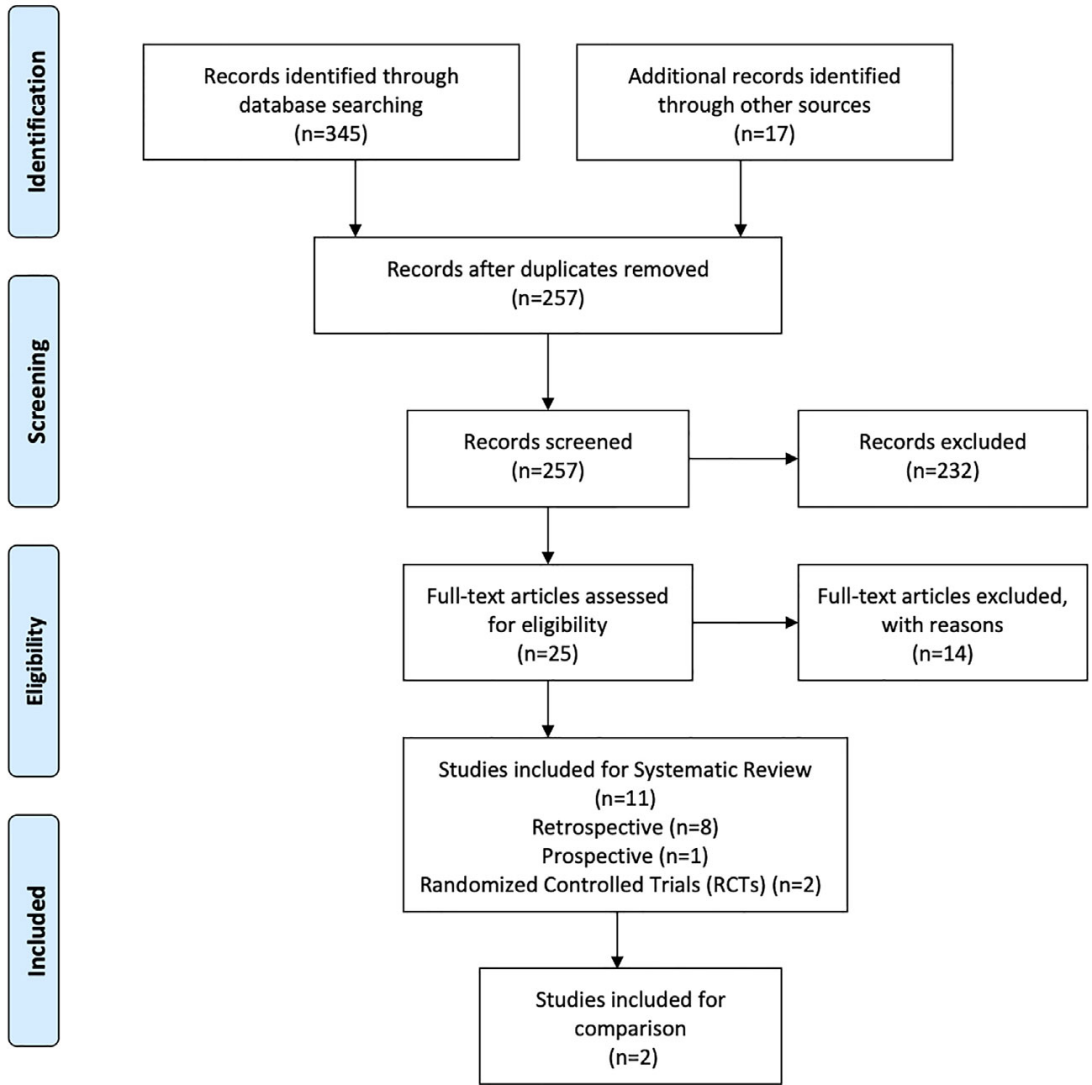
PRISMA flow diagram of research strategy and study selection.

**Figure 2:**
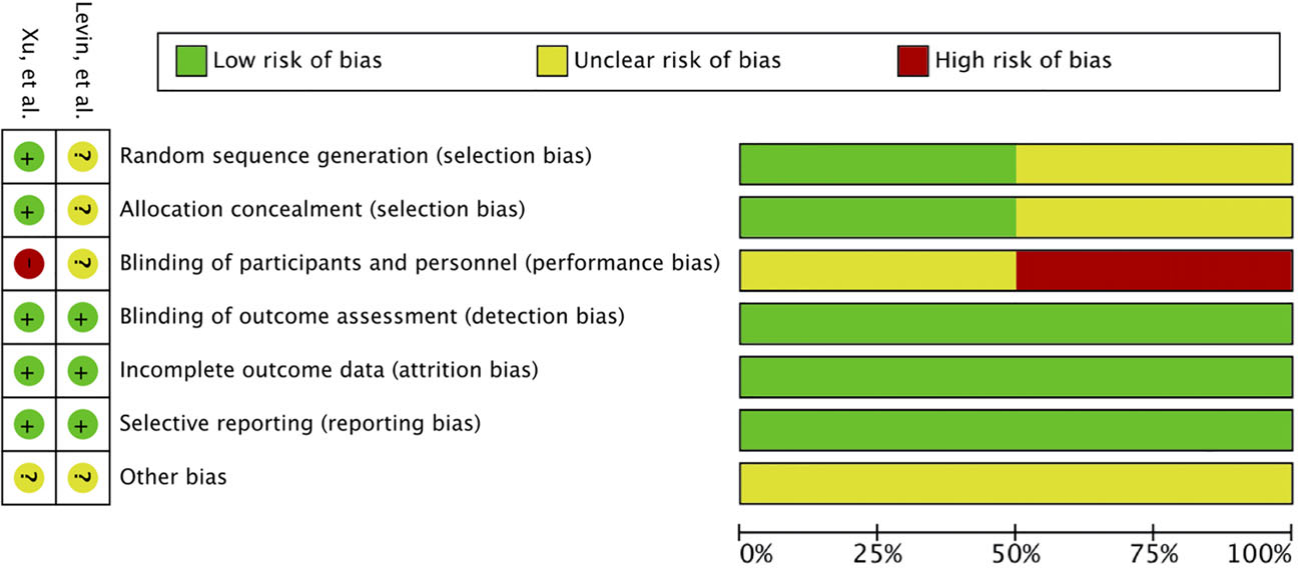
Risk of bias assessment of two randomized controlled trials.

A total of 345 studies were identified in the initial search. After vigilant screening of the titles and abstracts, 25 studies underwent full-text assessment. In accordance with the inclusion criteria, 12 studies (eight retrospective, two prospective, and two RCTs) were selected, including 236 patients who had developed RBN after undergoing radiotherapy and/or stereotactic radiosurgery (SRS) for the treatment of intracranial disease (22–33). The research strategy and study selection flowchart of this systematic review are presented in **Figure 1**. The two RCTs also included a comparison of bevacizumab with corticosteroid/placebo-based management of RBN (24, 32). The risk of bias assessment of these RCTs is shown in **Figure 2**.

### General Characteristics of Studies and Participants

**Figure 3:**
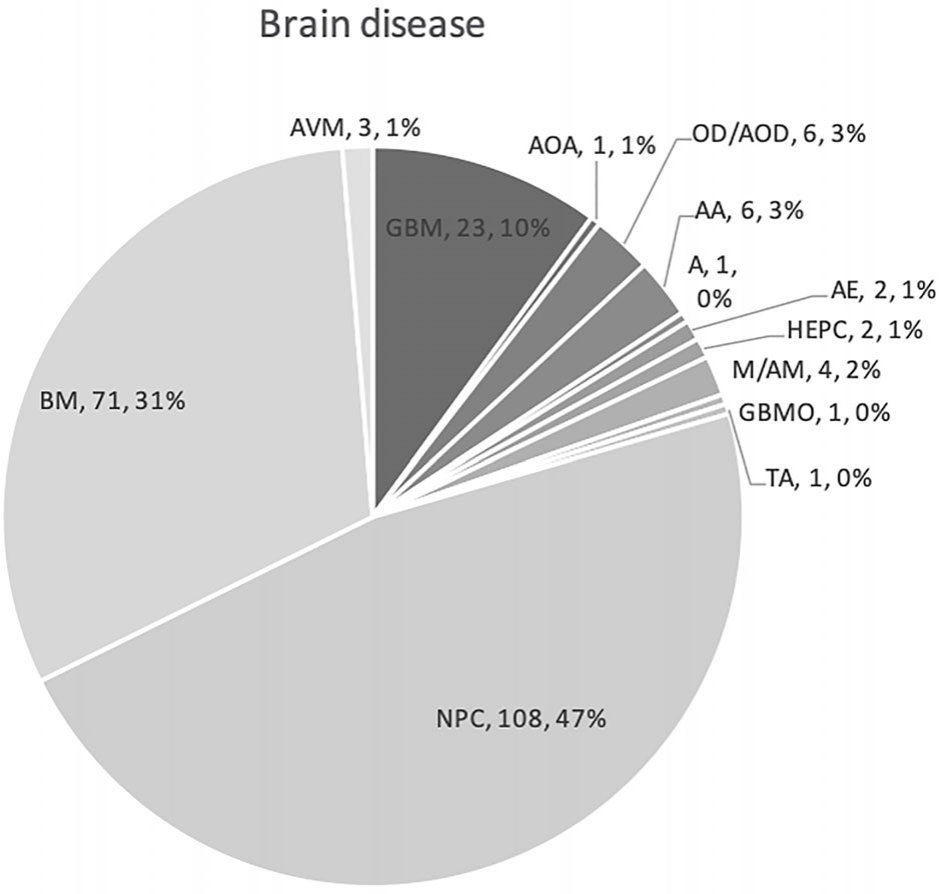
Graph showing various primary pathologies and metastatic brain disease for which cranial radiation was used. Abbreviations used indicates as follows: NPC, nasopharyngeal carcinoma; BM, brain metastases; GBM, glioblastoma; A, astrocytoma; AOA, anaplastic oligoastrocytoma; OD, oligodendroglioma; AOD, anaplastic oligodendroglioma; AA, anaplastic astrocytoma; GBMO, glioblastoma multiforme with oligodendroglial component; A, astrocytoma; HEPC, hemangiopericytoma; AE, anaplastic ependymoma; M, meningioma; AM, anaplastic meningioma; TA, tectal astrocytoma; and AVM, arteriovenous malformation.

**Figure 4:**
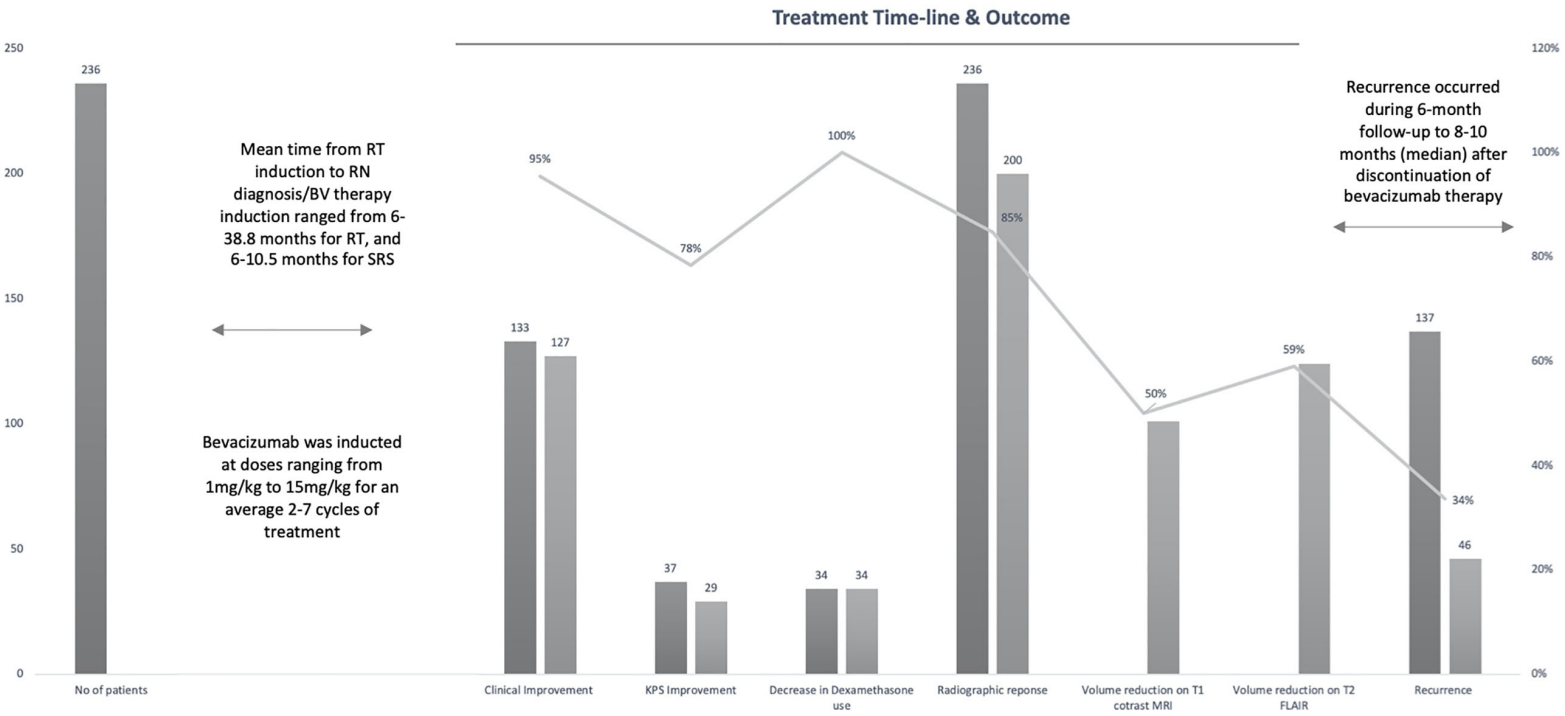
Graph showing timeline of entire study population from radiation therapy (RT) induction to radiation necrosis (RN) diagnosis/bevacizumab (BV) treatment to recurrence with main outcomes of interest.

The 12 studies included 236 patients with RBN who were treated with bevacizumab (22–33). Ninety-five of the participants were women (40%) and 141 were men (60%) (22–33). The patients’ intracranial diseases included various primary brain tumors, metastatic disease, nasopharyngeal carcinoma (NPC), and benign pathologies such as arteriovenous malformation with different frequencies, as presented in **Figure 3**. Patients with NPC were only included in two studies, but they comprised the largest disease group in the present study (n = 108), followed by those with brain metastasis (BM; n = 71) and glioblastoma (n = 23) (22–33). Conventional radiotherapy (external beam radiotherapy, intensity modulated radiotherapy, whole-brain radiotherapy, hypofractionated radiotherapy) and stereotactic radiotherapy were administered to 200 patients (83.7%) as the first course of treatment, while 31 patients (14.4%) received SRS as the first radiotherapy. Overall, 41 patients (19%) had undergone a second course/boost of radiation, of which 34 (83%) had received SRS. The diagnosis of RBN was predominantly confirmed by imaging findings on MRI, MRS, and PET scans. Biopsies were also provided in some studies (22–33). The diagnostic methods used in each study are listed in **Table 1**. The average time from induction of radiotherapy to RBN diagnosis or bevacizumab treatment ranged from 6 months to 38.8 months in patients who had received radiotherapy, and from 6.5 to 10.6 months in those who had undergone SRS. Various doses of bevacizumab were used, ranging from 1 mg/kg to 15 mg/kg every 2/3/4/6 weeks for an average of 2–7 cycles per study. The average follow-up time reported in the studies ranged from 8.1 months to 22.7 months. Detailed information about the characteristics of the studies, participants, and interventions is presented in **Table 1**. The timeline for overall management of the included patients, along with the main outcome results, is graphically presented in **Figure 4**.

**Table 1:** General characteristics of studies and participants.

Studies	Design & Period	Location	No. of Patients	Age(mean)	Male	Female	Basic histology	Radiation	RT to RN Diagnosis/BV Tx	RN Diagnosis	BV Dosage	No. of cycles	Follow Up
**Gonzalez et al. (** 22 **)**	Retrospective Sep 2005 - May 2006	The University of Texas M. D. Anderson Cancer Center, Houston, USA	8	54	4	4	AOA (1), AOD (1), AA (1), HEPC (1), GBM (4)	RT/SRS	38.8 months (RT) 9 months (SRS)	MRI	5 mg/kg q2 week OR 7.5 mg/kg q3 week	2-4	8.1 weeks
**Torcuator et al. (** 23 **)**	Retrospective 2006 - 2008	Henry Ford Hospital, Detroit, USA	6	48	3	3	A (1), AA (1), AE (1), GBM (3)	EBRT/FSRT/SRS	19.1 months (EBRT) 6.5 months (SRS/FSRT)	MRI, biopsy	10 mg/kg q2 week	6.8	5.9 months
**Levin et al. (** 24 **)**	RCT	University of Texas M. D, Houston, Texas, USA	7	47	5	2	AA (2), OD (2), HEPC (1), SCC (1)	RT	≥6 months	MRI, biopsy	7.5 mg/kg q2-3 week	4	10 months
**Wang et al. (** 25 **)**	Retrospective Mar 2010 - Jan 2012	Huashan Hospital, Fudan University, Shanghai, China	17	48	13	4	AA (1), AOD (1), GBM (7), M (1), BM (5) - (lung (2), colon (3)), AVM (1), FDB (1)	EBRT/SRS/FRST	34.3 (EBRT) 10.6 (SRS/FSRT)	MRI, MRS, PET	7.5 mg/kg q2 week	4 (median)	6 months
**Boothe et al. (** 26 **)**	Retrospective 3-year	Memorial Sloan- Kettering Cancer Center, New York, USA	11	58	4	7	BM (Breast (5), NSCLC (6))	SRS/WBRT	12.4 months	MRI, biopsy, PET	10 mg/kg q2w	6	101 days
**Furuse et al. (** 27 **)**	Retrospective Jan 2009 - Oct 2010	Osaka Medical College, Takatsuki, Osaka, Japan	11	57	6	5	BM (3), GBM (3), GBMO (1), AA (1), AM (3)	SRS/XRT/BNCT/ Proton/SRT	11 months	MRI, MET-PET	5 mg/kg q2w	3	14.4
**Yonezawa et al. (** 28 **)**	Prospective Nonrandomized Jun 2010 - Sep 2011	Kizawa Memorial Hospital, Minokamo, Japan	9	52.8	7	2	GBM (6) BM (2) - (lung) 2 AOD (1)	HFRT/WBRT/SRS/SRT	14.1 months	MRI, MET-PET	5 mg/kg q2w	6	
**Sadraei et al. (** 29 **)**	Retrospective Jul 2007 - Jun 2012	Cleveland Clinic, Cleveland, Ohio, USA	24	57	9	15	BM (17) - (lung (9), breast (4), rectal (1), melanoma (1), NSTC (1), FT (1)), GBM (2), AOD (1), AE (1), TA (1), AVM (2)	WBRT/SRS/Proton	16.2 months (RT) 9.8 months (SRS)	MRI, PET, biopsy	10 mg q2w OR 15 mg/kg q3w (11) 5 mg/kg q2w OR 7.5 mg/kg q3w (13)	7	8 months
**Zhuang et al. (** 30 **)**	Retrospective Jun 2011 - Dec 2014	Tianjin Cancer Hospital, Tianjin, China	14	56	6	8	BM (Lung (11), Breast (1), Lymphoma (1), Gastric cancer (1))	RT		MRI, PET, pathology	5 mg/kg q3-4w	3	12 months
**Li et al. (** 31 **)**	Retrospective	Guangzhou, China	50	50.7	35	15	NPC	RT/IMRT	≥6 months	MRI	5 mg/kg q2w	4	6 months
**Xu et al. (** 32 **)**	RCT Jul 2012 - May 2015	Guangzhou, China	58	49.3	38	20	NPC	RT/IMRT	≥6 months	MRI	5 mg/kg q2w	4	6 months
**Zhuang et al. (** 30 **)**	Prospective II CT Dec 2016 - Feb 2019	Tianjin Cancer Hospital, Tianjin, China	21	55 (median, range 43-70)	11	10	BM (lung (17), breast (2), kidney cancer (2))	SRT	17.6	MRI	1 mg/kg q3w	3	22.7
**This study**			236		141	95						2-7	

A, astrocytoma; AOA, anaplastic oligoastrocytoma; OD, oligodendroglioma; AOD, anaplastic oligodendroglioma; AA, anaplastic astrocytoma; GBM, glioblastoma; GBMO, glioblastoma multiforme with oligodendroglial component; A, astrocytoma; HEPC, hemangiopericytoma; AE, anaplastic ependymoma; M, meningioma; AM, anaplastic meningioma; SCC, squamous cell carcinoma; BM, brain metastases; AVM, arteriovenous malformation; FDB, fibrous dysplasia of bone; NSTC, non-seminomatous testicular cancer; FT, fallopian tube; TA, tectal astrocytoma; RT, radiotherapy; SRS, stereotactic radiosurgery; WBRT, whole-brain radiotherapy; XRT, X-ray radiotherapy; EBRT,electron beam radiotherapy; FSRT, fractionated stereotactic radiosurgery; HFRT, hypo-fractionated radiotherapy; SRT, stereotactic radiotherapy; BNCT, boron neuron capture therapy; NPC, nasopharyngeal carcinoma.

### Clinical Improvement

Clinical improvement was assessed in terms of improvement in neurological symptoms and signs, KPS score, and tapering of dexamethasone dosage.

#### Improvement in Neurological Symptoms/Signs

Resolution or improvement in neurological symptoms/signs and clinical outlook was reported in eight studies involving 133 patients who were radiographically responsive (23–26, 29, 30, 32). Overall, 127 patients (95%) demonstrated an improvement in clinical outcomes. Thirteen patients (9%) achieved a stable outlook, 106 (79.7%) showed improvement in symptoms, and eight (6%) experienced symptom resolution (23–26, 29, 30, 32). Further details are presented in **Table 3**. Two studies (n = 35) also analyzed improvement in symptomatic grades after bevacizumab therapy (30, 33). In a retrospective study by Zhuang et al. (n = 14), 10 patients (83.3%) had significantly reduced symptom grades after bevacizumab therapy (t = 5.657; p = 0.000) (30). In a prospective phase II trial by the same authors, 18 patients (90%) showed a significant decrease in the severity of their symptoms after bevacizumab induction (t = 5.657; p < 0.001) (33).

Bevacizumab was significantly better than corticosteroids, as revealed in the RCT conducted by Xu et al. (32), who reported significant clinical improvement in 36 patients (62.1%) treated with bevacizumab and in 23 patients (42.6%) treated with corticosteroids (p = 0.039). Levin et al. also observed clinical improvement in 100% of patients treated with bevacizumab, but in none who were treated with a placebo (0%) (**Table 2**) (24).

**Table 2:** Comparison of bevacizumab and corticosteroid-based treatment of radiation induced necrosis.

Studies	Levin et al.		Significance	Xu et al.		Significance
Treatment Groups	Bevacizumab	Placebo		Bevacizumab	Corticosteroids	
**Doasge**	intravenous bevacizumab at a dose of 7.5 mg/kg at 3-week intervals for 2 treatments	intravenous placebo at 3-week intervals for 2 treatments.		5 mg/kg intravenously every 2 weeks for up to 4 courses	methylprednisolone 500 mg/day intravenously for 3 consecutive days and then gradually tapered, followed by 10 mg/day oral prednisone, for 2 months in total	
**Characteristics**						
**No. of patients**	7	7		58	54	
**Age (mean)**	50	47.5		49.3	50.5	
**Male**	5	3		38	39	
**Female**	2	4		20	15	
**Main Outcomes**						
**Radiographic Response**	100%	0%	p = 0.0013	65.5%	31.5%	p < 0.001
**MRI Changes (volume reduction)**						
**T1 Gd enhancement**	63%	+17%	p = 0.0058	25.5%	5.0%	p = 0.027
**T2W FLAIR**	59%	+14%	p = 0.0149	51.8%	19.3%	p < 0.001
**Clinical Improvement**	7	0		36 (62.1%)	23 (42.6%)	p = 0.039
**Recurrence**	3			14	13	
**Adverse events**	6/11	0/7		41 (events)	52 (events)	

#### Improvement in KPS Score

Individual KPS scores were assessed in three studies involving 37 patients (25, 27, 28), all but eight of whom showed an increase in KPS score after treatment with bevacizumab (**Table 3**). The average increase in KPS score across the three studies ranged from 10 to 24.7 (25, 27, 28).

**Table 3:** Clinical improvement, average increase in KPS score, decrease in Dexamethasone use, and recurrence after bevacizumab treatment.

Characteristics/Studies	Gonzalez et al. (22)	Boothe et al. (26)	Wang et al. (25)	Furuse et al. (27)	Yonezawa et al. (28)	Torcuator et al. (23)	Levin et al. (24)	Sadraei et al. (29)	Zhuang et al. (30)	Li et al (31)	Xu et al. (32)	Zhuang et al. (30)	This study
**Clinical Improvement**													
No. of patients		11	16			6	7	23	12		38	20	133
Stable		3				3					7		13 (9%)
Improved		7	16			3	7	14	10		31	18	106 (79.7%)
Resolved								8					8 (6%)
													
**KPS Improvement**													
No. of patients			17	11	9								37
Improved			16	6	7								29 (78%)
Stable/decreased			1	5	2								8 (22%)
Average increase in KPS			24.7	11.6	10								
													
**Dexamethasone use**													
No. of patients	8	11	17			6	5						47
Decrease observed in patients	8	11	17			6	4						46 (97.8%)
Average decrease (mg)	8.6	8.7	9.4										
													
**Recurrence**													
No. of patients			16				7	23	13	38	38		135
Recurrence rate			1				2	4	10	15	14		46 (34%)

#### Decrease in Dexamethasone Dosage

Five studies evaluated the tapering of dexamethasone (**Table 3**) (22–26). Three studies involving 34 patients reported individual scores for changes in dexamethasone dosage (22, 25, 26). All patients registered a decrease in steroid use. The average decrease for the studies ranged from 8.6 to 9.4 mg (**Table 3**).

### Radiographic Response

All studies reported radiographic responses after bevacizumab use (22–33). Overall, 200 patients (84.7%) showed radiographic responses after the induction of bevacizumab, while 36 patients (15.3%) experienced radiographic progression (**Table 4**). Of the 36 patients with progressive disease, 32 had NPC (31, 32). Six studies including 52 patients revealed a 100% radiographic response (22–24, 26–28). Two studies involving NPC patients (n = 108) reported the lowest response rates (Li et al.: 76% [38/50]; Xu et al.: 65.5% [38/58]) (31, 32).

**Table 4:** Radiographic responses and MRI changes after treatment with bevacizumab.

Studies	No of patients	Radiographic responses	T1 Gd enhancement volume reduction (mean)	T2 FLAIR volumereduction (mean)
**Gonzalez et al. (** 22 **)**	8	100%	48%	60%
**Torcuator et al. (** 23 **)**	6	100%	79%	49%
**Levin et al. (** 24 **)**	7	100%	63%	59%
**Wang et al. (** 25 **)**	17	16 (94.1%)	54.9%	48.4%
**Boothe et al. (** 26 **)**	11	100%		65.5%
**Furuse et al. (** 27 **)**	11	100%	52.2%	56.7%
**Yonezawa et al. (** 28 **)**	9	100%	80% (p =0.01)	65% (p <0.001)
**Sadraei et al. (** 29 **)**	24	23 (95.8%)	48.1%	53.7%
**Zhuang et al. (** 30 **)**	14	13 (92.9%)	36%	59%
**Li et al. (** 31 **)**	50	38 (76.0%)		72.6% (p <0.001)
**Xu et al. (** 32 **)**	58	38 (65.5%)	25.5% (p <0.001)	51.8% (p <0.001)
**Zhuang et al. (** 30 **)**	21	20 (95.3%)	35%	74%
**This study**	236	200	Median 50%, Range 55% (26-80%)	Median 59%, Range 26% (48-74%)

The radiographic response was significantly better in patients receiving bevacizumab than in those given corticosteroids (24, 32). Levin et al. (n = 14) reported significantly better responses in patients treated with bevacizumab than in the placebo group (100% vs. 0%; p = 0.0013) (24). Xu et al. (n = 112) identified a similar significant response in patients treated with bevacizumab (65.5% vs. 31.5%; p < 0.001) (32).

#### MRI Changes

All 12 studies reported volume reduction on T1 Gd enhancement and/or T2 FLAIR MRI images (22–33). A median reduction of 50%, with a range of 26%–80%, was observed on T1 Gd enhancement MRI in 10 studies (22–26, 28–30, 32, 33) (**Table 4**). Similarly, T2 FLAIR MRI showed a median volume reduction of 59%, with a range of 48%–74%, in 12 studies (22–33). The volume reduction in contrast studies and flair images in all studies are illustrated in **Table 4**.

Several studies have also reported significant MRI volume reduction after bevacizumab therapy initiation (28, 30–32). In patients with NPC, significant volume reduction was seen in both MRI images after bevacizumab treatment (T1: 25.5%, p < 0.001; T2: 51.8%, p < 0.001) (32). Yonezawa et al. (n = 9) also identified a significant reduction in both MRI images after bevacizumab induction (T1: 80%, p = 0.01; T2: 65%, p < 0.001) (28). The mean reduction in edema index was significant in 64.3% of patients in the study by Zhuang et al. (p = 0.002) (30). Similarly, a study by Li et al. (n = 38) reported a significant mean reduction in volume under T2-weighted FLAIR MRI (72.6%; p < 0.001) (31).

Xu et al. reported that 38 of 58 patients (65.5%) in the bevacizumab group had a mean 25.5% reduction in edema volume on T1 post-Gd MRI and a mean 51.8% reduction on T2-weighted FLAIR images (32). In contrast, the corticosteroid group achieved reductions of only 5.0% and 19.3% on T1 Gd enhancement and T2-weighted FLAIR MRI, respectively (32). This difference was significant (**Table 2**). Levin et al. reported a mean T2 FLAIR edema volume reduction of 59% across all patients (5/5; 100%) randomized to receive bevacizumab (24), while all patients in the control group (7/7 patients; 100%) showed a mean increase of 14% in T2 FLAIR edema volume (p = 0.0149; **Table 2**). Bevacizumab treatment reduced the T1-enhancement volume by 63%, while the control group saw a 17% increase (p = 0.0058) (24).

### Necrosis Recurrence

Overall, six studies involving 135 patients reported the recurrence rates following bevacizumab therapy for RBN (24, 25, 29–32). In total, 46 responding patients (34%) had recurrence after showing improvement with bevacizumab therapy (**Figure 4**). The highest recurrence rate was observed in patients with brain metastasis (10 of 14 patients) (30). Ten patients were reported to have recurrence during a median follow-up time of 10 months (1.2–38 months) after discontinuation of bevacizumab therapy. Three of the five patients responded to retreatment with bevacizumab (30). Patients with NPC who responded to bevacizumab treatment also had a high recurrence rate (Li et al.: 15/38 [39.5%]; Xu et al.: 14/38 [36.8%]) (31, 32). During the 6-month follow-up, 14 of 58 patients had recurrence (32). Four patients (17.3%) experienced recurrence in the study by Sadraei et al. (29). The median time of RBN recurrence was 8 months (range: 6–16 months) in these four patients who had neurological deterioration after initial response to bevacizumab (29). All four patients responded to bevacizumab rechallenge, and one experienced RBN recurrence for the third time after 4 months of treatment (29). The patient responded and showed clinical improvement with a third bevacizumab therapy (29). One patient in the study by Wang et al. (n = 17), and two randomized to receive bevacizumab in the study by Levin et al., also had RBN recurrence. No other studies mentioned recurrence in their assessments (22, 23, 26–28, 33).

The recurrence rate of bevacizumab did not differ significantly from that of corticosteroids or placebo. Xu et al. reported that 14 of 58 patients in the bevacizumab group and 13 of 54 patients in the control group experienced necrosis recurrence during the 6-month follow-up (32). Levin et al. reported that two of seven patients originally randomized to receive bevacizumab treatment had RBN recurrence (24). Comparison with the placebo group could not be carried out in that study as all patients in the placebo group had progression upon treatment initiation, and six of them were crossed over to the bevacizumab group. Overall, 25% (3/12) of recurrences were observed in patients from the placebo group, including the crossed over patients (24).

### Adverse Events

Adverse events related to bevacizumab were reported in seven of the included studies (23–25, 28–30, 32). Most of the side effects were grade 1 or 2. The most common symptoms were hypertension, fatigue, proteinuria, and ischemic changes (23–25, 29, 30, 32). Two grade 3 events were reported: pulmonary embolism and ischemic stroke (29, 32). Other less frequent side effects are presented in **Table 5**.

**Table 5:** Adverse events reported with bevacizumab treatment.

Studies	Patients	Symptoms
**Torcuator et al. (** 23 **)**	1 (17%)	Fatigue (1)
**Levin et al. (** 24 **)**	6 (55%)	Aspiration pneumonia (1), pulmonary embolus secondary to DVT (1), superior sagittal sinus thrombosis (1), ischemic changes due to small vessel thrombosis (3)
**Wang et al. (** 25 **)**	3 (18%)	Grade 2 AEs: hypertension (1), fatigue (1), proteinuria (1)
**Yonezawa et al. (** 28 **)**	3 (33%)	Grade 1 or 2: anemia, leukopenia, neutropenia, and lymphocytopenia
**Sadraei et al. (** 29 **)**	7 (29%)	Grade 2 or less: hypertension, fatigue, urinary tract infection, and proteinuria (6). Grade 3: pulmonary embolism (1)
**Zhuang et al. (** 30 **)**	2 (14%)	Mild allergy, hypertension
**Xu et al. (** 32 **)**		Grade 1 or 2: hypertension (12), fatigue (7), infection (4), hemorrhage (4), insomnia (3), headache (3), rash (3), fever (2), blurred vision (1), hyperglycemia (1). Grade 3: Ischemic stroke (1).

Levin et al. reported no adverse events in 7/7 patients who received placebo, but that 6/11 patients receiving bevacizumab had adverse events, including the six patients who had crossed over to the bevacizumab group (24). Hence, no comparison can be made based on these data. Xu et al. indicated that the two groups had similar rates of adverse events (32). Patients receiving bevacizumab experienced 41 adverse events, whereas the corticosteroid group reported 52 events.

### Improvement in Cognitive Function

Improvement in cognitive function was only assessed in RCTs comparing bevacizumab with placebo/corticosteroids (24, 32). Between-group differences in the change scores of medium to large effect size (Cohen’s d ≥ 0.5) revealed that bevacizumab-treated patients had greater improvement than the placebo group in a learning trial (Hopkins Verbal Learning Test-Revised [HVLT-R] total recall) (Cohen’s d = 0.59; p = 0.26) and a delayed recognition memory trial (HVLT-R delayed recognition; Cohen’s d = 0.92; p = 0.08), as well as improved symptom severity ratings (MD Anderson Symptom Inventory [MDASI]-Severity; Cohen’s d = 0.68; p = 0.19). On the other hand, Levin et al. indicated that all bevacizumab-treated patients (11 patients; 100%) exhibited worse performance on the memory measure in the delayed free recall trial (HVLT-R delayed recall), which had a low effect size (Cohen’s d = -1.05), whereas all placebo-treated patients (seven patients; 100%) showed improvement (24). Independent sample t-tests on change scores demonstrated that this difference was of borderline statistical significance (p = 0.052). All bevacizumab-treated patients (11 patients; 100%) also reported experiencing greater interference in their everyday activities due to their symptoms (MDASI-Interference) in comparison to the placebo-treated patients (7/7 patients, 100%; Cohen’s d = -0.81; p = 0.13).

The Montreal Cognitive Assessment (MoCA) scale was used to assess cognitive function by Xu et al. (32). At baseline, 21 of 58 patients (36.2%) in the bevacizumab group and 16 of 54 patients (29.6%) in the corticosteroid group had MoCA scores < 26, with no significant difference between the groups. After 2 months of treatment, 28 of 58 patients (48.3%) in the bevacizumab group had improved MoCA scores, with a mean score change of 0.71 ± 2.02. On the other hand, 10 of 54 patients (18.5%) in the corticosteroid group showed improved MoCA scores, with a mean change of 0.00 ± 0.91 (p = 0.028).

## Discussion

RBN is a late complication that can follow radiotherapy in all or parts of the brain (34, 35). Incidence rates of 5%–59% have been reported (7, 36–41). The incidence of RBN is affected by multiple factors, including the radiotherapy modality, the dose of radiation, the dose of fraction, the adjuvant treatment received (chemotherapy, immunotherapy, and targeted therapy), and the diagnostic methods used (7, 36–44). RBN is a dose-limiting factor in SRS, and an incidence rate of approximately 10% to 25% has been reported after SRS, depending on the length of follow-up (7, 36, 37, 42, 45, 46). Diagnostic methods have also defined contrast incidence rates (41, 43, 44). An incidence rate of 7% was observed in studies where pathological confirmation or temporal resolution was mandatory (43). Minniti et al. reported an RBN incidence rate of 24% (14% symptomatic, 10% asymptomatic). They relied on imaging features for RBN diagnosis, such as contrast enhancement, absence of progression for > 4 months, and reduced perfusion on dynamic MRI sequences (44). Therefore, determining the incidence rate of RBN is complex and requires the assessment of various contributing factors.

The treatment options for RBN include corticosteroids, bevacizumab, hyperbaric oxygen therapy, surgical resection, and laser-induced thermal therapy (35, 38, 39, 47–50). Corticosteroids are the first-line treatment for RBN as they effectively inhibit the pro-inflammatory response that propagates necrosis and reduces the leakiness of the blood-brain-barrier (34, 47). Symptomatic relief is achieved *via* edema reduction; however, long-term use results in steroid myopathy, osteopenia, gastric ulcers, glucose intolerance, and iatrogenic Cushing’s syndrome (48). The rationale for using hyperbaric oxygen therapy to treat cerebral radiation necrosis is that increasing the oxygen concentration stimulates angiogenesis and restores the blood supply to the necrotic lesion, thereby promoting healing (38, 49–53). However, evidence for the efficacy of this approach mainly relies on case reports, with no class I evidence (49–52). Simultaneously, this treatment is expensive, longer lasting, and requires special facilities, so it is less desirable and feasible in clinical practice. Surgery is also an important method for managing progressive resectable radiation necrosis lesions as it can relieve the effects of the mass itself (35, 38, 39, 53, 54). It also allows tissue diagnosis and can be used to rule out tumor progression by biopsy (35, 38). However, even after surgical resection, brain edema may persist for a few weeks and require close monitoring (35, 38, 53, 54). Recently, laser interstitial thermal therapy and anti-VEGF targeted inhibitors have been used in addition to surgery to treat RBN (38, 55). Various studies have shown clinical improvement and enhanced progression-free survival and overall survival rates with laser interstitial thermal therapy (56–58). Nevertheless, treatment guidelines in patients with RBN are inconsistent, so further class I evidence is required, including RCTs, to establish the role of each treatment modality.

Recently, the efficacy of bevacizumab in RBN treatment has been investigated (12–33). Several case reports and case series have reported a positive response to bevacizumab treatment (12–19). We systematically reviewed the literature for studies that clinically evaluated patients with RBN who were treated with bevacizumab. We obtained 12 studies, with a total population of 236 patients (22–33). The radiographic response to bevacizumab was nearly 97%, excluding patients with NPC (22–30), who had a 70% response to bevacizumab treatment (31, 32). The responding patients demonstrated edema volume reduction under T1 Gd enhancement (median: 50%, range: 26%–80%) and T2-weighted FLAIR MRI images (median: 59%, range: 48%–74%) (22–33). Some of these studies found that the volume reduction was significant on both MRI images after bevacizumab treatment (28, 30–32). Furthermore, both RCTs demonstrated that the reduction induced by bevacizumab treatment was significantly superior to that induced by corticosteroids (24, 32).

The radiographic response was correlated with clinical improvement, as 91% of the patients with radiographic response had either stabilized clinical outlook or improved and resolved neurological symptoms and signs (23–26, 29–33). The improvement could also be reflected in the increase seen in the individual KPS scores of 29 patients (79%) after bevacizumab treatment (25, 27, 28). A significant decrease in the use of dexamethasone was also observed in each individual patient in the five studies that reported this variable (22–26).

Although the literature is limited in this regard, several case reports and case series have yielded similar efficacy data (12–19, 59–61). One systematic review assessed 16 studies, including seven single-case reports and a total of 71 unique RBN cases reported between 2007 and 2012. That study revealed similar efficacy data for bevacizumab in the management of RBN (15). A radiographic response of 97% in 79% of patients showed improvement in performance status. The KPS improvement was 10 points (range: 0–40), and a 6-mg (range: 0–24 mg) decrease in dexamethasone use was noted. Median decreases of 63% and 59% in T1 contrast enhancement and T2/FLAIR signal abnormality, respectively, was revealed after treatment (15). A recently published case report of four ALK-positive lung cancer patients with RBN revealed that all of the patients had seen a decrease in RBN and that three had experienced symptom improvement after bevacizumab (15 mg/kg every 3–4 weeks) was added to their ALK-TKI therapy (59). Administration of lower bevacizumab doses (2 x 7.5 mg/kg every 3 weeks) also resulted in complete resolution of physical signs in two patients (one with metastatic lung cancer and another with NPC), as well as a transient improvement in signs and symptoms in two other patients with primary brain tumor (19). A case report of two melanoma patients with brain metastasis and symptomatic RBN showed clinical improvement and discontinuation of dexamethasone after initial doses of 5 mg/kg every 2 weeks (18). Bevacizumab has also shown responses in patients with steroid-refractory RBN (16, 17, 60, 61). A case series of four patients with high-grade glioma, who had developed cerebral edema due to tumor progression or radiation necrosis and were either not responding to corticosteroids or were not candidates for surgical debulking, showed rapid responses to bevacizumab treatment (17). Radiographic changes were observed on both T1 and T2 MRI images in all four patients, and three of them also showed a reduction in dexamethasone use (17). Similarly, in a retrospective study, bevacizumab was administered to 17 patients with radiation-induced brain edema refractory to steroids (61). The bevacizumab was administered at an increasing dose every 2 weeks (5–7.5–10 mg/kg). Approximately 82% of the patients showed clinical improvement, and the majority showed improvement after the third dose (61). In our systematic review, some patients did not show any clinical improvement or experienced symptomatic worsening and progression. The medical literature reveals similar examples. In a study by Gronier et al., no clinical improvement was observed in any of three patients with malignant brain tumors after bevacizumab therapy (10 mg/kg per month) (62). One patient experienced lymphopenia after one perfusion of bevacizumab; the other developed a transient ischemic attack and a corneal ulcer (62).

With regards to neurological function, formal neurocognitive testing revealed a mixed pattern of findings from both objective tests of neurocognitive function and self-reported measures of symptoms, as reported by Levin et al. (24). There was a trend toward improvement in aspects of learning and memory after 6 weeks of therapy, suggesting improved memory encoding and retention despite increased deficits in memory retrieval. This pattern suggests dysfunction in the frontal subcortical systems. However, these findings might also be explained by the small sample size of both groups. On the other hand, Xu et al. reported a greater improvement in bevacizumab-treated patients, as assessed using the MoCA scale (32). These outcomes indicate that bevacizumab treatment is safe and that it enhances neurocognitive functions. Similarly, two case series reports showed that bevacizumab can alleviate neurocognitive deficits (19). In the first, administration of bevacizumab (500 mg twice) sufficiently improved memory loss and reversed lower extremity weakness. In the second, the Mini-Mental State Examination score was elevated after administration of 400 mg bevacizumab twice. In both cases, improvements were evident on MRI imaging.

In many studies, bevacizumab has elicited a therapeutic response and clinical improvement at any prescribed dose ranging from 1 mg/kg to 15 mg/kg (12–19, 22–33, 59–61). The duration between each cycle has also varied, ranging from every 2 weeks to every 6 weeks. Zhuang et al. suggested that bevacizumab efficacy is associated with its anti-angiogenic effects rather than the dose (33). A previous systematic review recommended an initial dose of 7.5 mg/kg every 2 weeks for four cycles to treat patients with RBN (15). Tripathi et al. suggested that, to achieve the best response, an average of seven cycles (range: 5–27) of bevacizumab should be administered (61). Our study also indicated that bevacizumab should be administered for up to seven cycles on average (**Table 1**) (22–33). However, prolonged treatment should be avoided, as paradoxical phenomena have also been reported (16, 63). In a case report, exacerbation of RBN was noted after the initial response to bevacizumab (16). The authors speculated that there had been initial cerebral edema reduction, but that prolonged treatment caused over-pruning of at-risk blood vessels within the radiation field (16). Over-pruning likely leads to vascular insufficiency, which can exacerbate hypoxia and necrosis (63).

In the present study, 34% of the radiographically responsive patients experienced recurrence, based on the results of six studies (24, 25, 29–32). The underlying cause of recurrence was not identified in any of the studies. Li et al. indicated that the duration between radiotherapy and bevacizumab intervention and the duration between radiotherapy and RBN diagnosis were predictive factors for the recurrence of RBN after bevacizumab treatment (31). In another study, recurrence of RBN after bevacizumab treatment was correlated with the time since initial bevacizumab withdrawal (64). As with the primary diagnosis of RBN, RBN recurrence is difficult to differentiate from a tumor (39, 65–68). In a study of 14 patients with brain metastasis, 10 of the 13 responsive patients (76.9%) experienced a recurrence of RBN during follow-up (30). Similarly, two studies involving patients with NPC reported a high recurrence rate (36%–38%) (31, 32). Although the two studies differed in research design (one retrospective, one RCT), the results were very consistent (31, 32). In the comparative RCT, both patients receiving bevacizumab and corticosteroids experienced similar recurrence rates (32), indicating that RBN recurrence may not prove failure of bevacizumab treatment. Nonetheless, no pathological confirmation was sought to confirm the RBN recurrence in these studies (30–32). In a case report, a 55-year-old man experienced re-enlargement of RBN after 8 months of bevacizumab therapy (69). This re-enlargement was attributed to the recurrence of lung cancer, as histopathological analysis of the resected specimen revealed large necrotic areas with viable tumor cells (69). Hence, an accurate recurrence rate could only be determined by pathology, which should be further examined in larger, comprehensively organized trials.

Retrospective studies reported side effects ranging in 14%–33% of patients after bevacizumab treatment (23, 25, 28–30). The side effects were mostly grade 1 or 2, with only one grade 3: pulmonary embolism (23, 25, 28–30). A previous study also reported a similar low percentage of grade 3 or 4 toxicities (2.3%) (64). The two RCTs showed an increased number of side effects, but they were low grade (24, 32). In the study by Levin et al., adverse events occurred in 55% of patients (6/11) in the bevacizumab group; however, this number also included crossed-over placebo patients (24). Similarly, Xu et al. found that bevacizumab treatment caused no more side effects than corticosteroid-treatment (32). Therefore, bevacizumab may not increase the risk of serious side effects in patients with RBN.

Bevacizumab is used to treat RBN because RBN tissues have elevated levels of VEGF (70, 71). However, circulating levels of VEGF did not predict the therapeutic efficacy of bevacizumab in a study by Li et al. (31). The pre- and post-bevacizumab VEGF levels in responsive and non-responsive patients revealed no differences (68.0 ± 13.1 pg/mL vs. 68.1 ± 21.3 pg/mL; p = 0.950). Moreover, VEGF levels did not predict recurrence, as patients with recurrence showed no difference in serum VEGF levels from those without recurrence (66.8 ± 11.1 pg/mL vs. 69.2 ± 14.4 pg/mL; p = 0.931) (31).

Several factors limited our analysis. Firstly, most of the studies were retrospective in nature and contained few patients (22, 23, 25–27, 29–31). Retrospective studies are prone to selection bias, recall bias, and misclassification bias, and they are subject to confounding (72). Furthermore, patients had different conditions for undergoing radiotherapy; radiation modalities and doses also differed greatly among the studies and patients (22–32). The follow-up after bevacizumab therapy also varied and was limited (22–32). The patients were most often diagnosed using radiological evaluation in the included studies (22–33). Publication bias may also have been present, as patients responding to bevacizumab were more likely to be included in the studies (22, 23, 25–27, 29–31). To assess comparative outcomes, only two RCTs were available. Even though it was classified as class I evidence, the study by Levin et al. comprised only 14 patients and a high crossover from the placebo group. The second RCT was downgraded one level as it was open-label; it was then further downgraded due to concerns regarding detection bias and inadequate blinding in the trial (32).

## Conclusions

In conclusion, patients with RBN respond to bevacizumab treatment. Bevacizumab improves the neurological symptoms in patients with RBN, as well as performance as measured using the KPS scale. Patients ultimately either discontinued or tapered the use of dexamethasone. Although several studies were retrospective, their efficacy data were consistent with the RCT results. The improvement was also evident on MRI images, with a significant reduction in edema volumes. The results of the two RCTs suggested that bevacizumab treatment had superior efficacy than placebo or corticosteroids. Importantly, bevacizumab could improve neurocognition. Currently, a randomized controlled trial is ongoing, comparing bevacizumab plus corticosteroids with corticosteroids plus placebo. This trial which will certainly provide more evidence for the comparative efficacy of bevacizumab (NCT02490878). There were no serious side effects associated with bevacizumab administration. The safety profile was comparable to that of the corticosteroids. More well-designed and larger RCTs are required to fully establish the role of bevacizumab in RBN treatment.

## Data Availability Statement

The original contributions presented in the study are included in the article/supplementary material. Further inquiries can be directed to the corresponding authors.

## Author Contributions

GL, MK, and ZZ provided manuscript writing, data searched and data analysis. All authors corrected and proofed the final text. All authors contributed to the article and approved the submitted version.

## Funding

The Natural Science Foundation of Shenzhen (No. JCYJ20170307095828424) and Shenzhen Health and Family Planning System Research Project (No. SZBC2017024) were providing support for this work.

## Conflict of Interest

The authors declare that the research was conducted in the absence of any commercial or financial relationships that could be construed as a potential conflict of interest.
